# Placenta‐derived exosomal miR‐135a‐5p promotes gestational diabetes mellitus pathogenesis by activating PI3K/AKT signalling pathway via SIRT1

**DOI:** 10.1111/jcmm.17941

**Published:** 2023-09-04

**Authors:** Qiuyu Zhang, Xu Ye, Xia Xu, Jianying Yan

**Affiliations:** ^1^ Department of Obstetrics and Gynecology, Fujian Maternity and Child Health Hospital Affiliated Hospital of Fujian Medical University Fuzhou China

**Keywords:** exosome, gestational diabetes mellitus, miR‐135a‐5p, PI3K/AKT, placenta, SIRT1

## Abstract

Most people are aware of gestational diabetes mellitus (GDM), a dangerous pregnancy complication in which pregnant women who have never been diagnosed with diabetes develop chronic hyperglycaemia. Exosomal microRNA (miRNA) dysregulation has been shown to be a key player in the pathophysiology of GDM. In this study, we looked into how placental exosomes and their miRNAs may contribute to GDM. When compared to exosomes from healthy pregnant women, it was discovered that miR‐135a‐5p was elevated in placenta‐derived exosomes that were isolated from the maternal peripheral plasma of GDM women. Additionally, we discovered that miR‐135a‐5p encouraged HTR‐8/SVneo cell growth, invasion and migration. Further research revealed that miR‐135a‐5p activates HTR‐8/SVneo cells' proliferation, invasion and migration by promoting PI3K/AKT pathway activity via Sirtuin 1 (SIRT1). The transfer of exosomal miR‐135a‐5p generated from the placenta could be viewed as a promising agent for targeting genes and pertinent pathways involved in GDM, according to our findings.

## 1 INTRODUCTION

Atypical glucose metabolism is referred to as gestational diabetes mellitus (GDM) when it is discovered for the first time during pregnancy. The prevalence of GDM increases with gestational weight and age, causing serious adverse effects in both the mother and offspring. Although symptoms gradually improve after delivery, GDM is related to later development of type 2 diabetes and an increased risk of cardiovascular disease, which may continue to affect maternal and infant health.^1^

As one of the most significant pathogenic factors in GDM development, insulin resistance impairs pancreatic β‐cells and disrupts the compensatory ability to regulate blood sugar.^2^ GDM is believed to originate from gene mutations and/or an imbalance in placental hormone regulation.^3^ A large number of signalling pathways have been shown to participate in GDM development, such as the AMPK, FOXO and WNT signalling pathways.^4^, ^5^, ^6^ Therefore, functional biomarkers associated with lipid and glucose metabolism need to be identified.

Exosomes are extracellular vesicles that are released by a variety of cells. They contain a wide range of substances, such as cytosolic and cell surface proteins, lipids, metabolites, DNA and RNA. Notably, exosomes are highly stable in body fluids because of their specific lipid bilayer membrane.^7^ They are selectively targeted to specific cells, tissues, or organs and transfer their cargo to modify the associated bioactivity, which contributes to the development of various diseases, such as cancer progression, immune responses, cardiovascular diseases and metabolic complications.^8^ These exosome‐mediated responses are critical for increasing bioavailability and reducing negative effects, and they can either promote or prevent disease.

Recently, plenty of scholars have demonstrated that exosomal microRNA (miRNA) plays an important role in maintaining GDM pancreatic β‐cell function and insulin excretion. As placental exosomes released into maternal circulation can be detected with aberrant miRNAs after 11 weeks of gestation, pregnancy‐associated complications can be diagnosed by detecting placenta‐derived exosomal miRNAs during early pregnancy.^9^

However, nothing is known about how exosomal miRNAs from the placenta influence GDM development. The current study aimed to profile the differentially expressed miRNAs in circulating exosomes of GDM patients and investigate the possible targeted genes and relevant pathways involved in GDM.

## 2 METHODS

### 2.1 Patients and samples

From June 2021 to December 2021, pregnant women who completed routine obstetric examinations and caesarean births at the Obstetrics Department of Fujian Maternal and Child Health Hospital were chosen. According to the guidelines of the International Association of Diabetes and Pregnancy Study Groups, GDM was identified using a three sample 75 g oral glucose tolerance test (OGTT) at 24–28 weeks. The following were the requirements for study inclusion: fasting glucose ≥5.1 mmoL/L, 1 h glucose ≥10.0 mmoL/L and 2 h glucose ≥8.5 mmoL/L. Multiple births, high blood pressure, pre‐pregnancy diabetes (or any other conditions impacting blood glucose levels) and severe cardiac, hepatic and renal failure were the exclusion criteria. According to the outcomes of the OGTT, the patients were split into two groups: a control group (*n* = 30) and a GDM group (*n* = 30). For additional investigation, samples of placenta and fasting blood were taken and kept at −80°C. Each participant provided their informed consent, and the study methodology was approved by the Fujian Provincial Maternal and Child Health Hospital's ethics committee in accordance with the Declaration of Helsinki.

### 2.2 Microarray data

The terms “microRNA or miRNA” and “diabetes” were used in a microarray search in the Gene Expression Omnibus (GEO) database (http://www.ncbi.nlm.nih.gov/geo/). The entry type was limited to the ‘series’, and the organism was filtered by ‘Homo sapiens.’ The following were the inclusion requirements: (1) Human serum, plasma or placental tissue were the chosen samples, and (2) both the control and GDM groups were included. A download of the gene expression microarrays followed. From the raw gene expression microarray data, each gene expression dataset was retrieved. Finally, the following analyses were chosen because they satisfied the criteria: GEO Series 98,043 (GSE), GSE94649, GSE97123 and GSE148961.

### 2.3 Data process

Using the GEO2R analytical tool (https://www.ncbi.nlm.nih.gov/geo/geo2r/), which is offered by the GEO database, differentially expressed miRNAs (DEMi) were screened with *p*‐value 0.05 and | log2FC| > 0.5 as the cutoff criteria. The common DEMi were displayed as a Venn diagram, and each dataset was displayed as a volcano plot.

### 2.4 Functional and pathway enrichment analysis

An online miRNA pathway analysis tool is DIANA mirPath v.3 (https://dianalab.e‐ce.uth.gr/html/mirpathv3/index.php?r=mirpath). It performs pathway enrichment analysis, retrieves experimentally confirmed miRNA target genes from the DIANA Tar Base (https://dianalab.e‐ce.uth.gr/html/diana/web/index.php?r=tarbasev8) and predicts miRNA targets (CDS or 3′‐UTR regions) provided by the DIANA‐microT‐CDS algorithm. The cutoff for statistical significance was *p* = 0.05. The outcomes were validated and illustrated using PathCards and Reactome (https://pathcards.genecards.org/ and http://reactome.org/, respectively).

### 2.5 Cell culture and transfection

The American Type Culture Collection (ATCC, Manassas, VA, USA) provided the human trophoblast HTR‐8/SVneo cells, which were then purchased and cultured in a high‐glucose (4.5 g/L) Dulbecco's Modification of Eagle's Medium solution with 10% Foetal Bovine Serum at 37°C in a humid environment with 5% CO_2_. GenePharma Co., Ltd. supplied the miR‐135a‐5p mimics, miR‐135a‐5p inhibitor, miR‐135a‐5p mimics‐NC and miR‐135a‐5p inhibitor‐NC. The transfection agent was Lipofectamine 2000 (Invitrogen). RNA and total protein were collected from the transfected cells after 48 h to assess the effectiveness of the transfection.

### 2.6 Exosome isolation

Utilizing the exoEasy Maxi Kit from Qiagen (Qiagen, 76,064), exosomes were recovered from plasma. Buffer XBP was combined with pre‐filtered plasma (particles larger than 0.8 μm were excluded) and coupled to an exoEasy membrane affinity spin column. Buffer XWP was used to wash the bound exosomes, and Buffer XE, 400 μL, was used to elute them. The exosomes were then prepared for additional analysis.

### 2.7 Transmission electron microscopy

Exosomes were filtered through a formaldehyde/carbon‐coated copper grid and then negatively stained with 1% phosphotungstic acid for 3 min at room temperature. The copper grid was then dried at room temperature after being cleaned with ddH_2_O three times. The stained exosomes were observed using a JEM‐1400 electron microscope from JEOL Ltd.

### 2.8 Nanoparticle tracing analysis

Exosome sizes and concentrations were analysed using nanoparticle tracking analysis. With the help of a ZetaView PMX 110 (Particle Metrix, Meerbusch, Germany), fresh exosomes diluted in PBS were found. The software ZetaView was used to process and analyse the collected images.

### 2.9 Western blot analysis

The expression of exosome‐specific biomarkers and target proteins, such as placental alkaline phosphatase (PLAP), Sirtuin 1 (SIRT1), CD9 antibody and CD63 antibody, was examined by Western blotting (ImmunoWay Biotechnology). All proteins were extracted from tissue samples or cells using the radioimmunoprecipitation assay (RIPA) lysis buffer (ThermoFisher Scientific) and 1% protease inhibitor cocktail (Sigma‐Aldrich). Using 10% sodium dodecyl sulfate‐polyacrylamide gel electrophoresis (SDS‐PAGE), 20 μg of total protein was separated and then deposited onto a polyvinylidene fluoride membrane. Membranes were incubated with primary antibodies for an overnight period at 4°C after being blocked with 5% non‐fat milk for 2 h at room temperature. Horseradish peroxidase‐conjugated secondary antibodies that were appropriate were utilized. The Western Blotting Luminol Reagent (Bio‐Rad) was used to identify proteins. A chemiluminescence reaction (ECL Prime, GE Health Life Sciences) was produced using goat anti‐rabbit or goat anti‐mouse secondary antibodies that were horseradish peroxidase conjugated (ImmunoWay Biotechnology Company). The ImageJ program (NIH, USA) was used to examine the density of the bands.

### 2.10 Haematoxylin and eosin (H&E) staining

After being treated in a 4% paraformaldehyde solution, the placental tissue was embedded in paraffin wax. Haematoxylin and eosin (H&E) staining was applied to 5‐μm‐thick slices of the collected tissues. A light microscope was used to examine the sections.

### 2.11 Reverse transcription polymerase chain reaction (RT‐qPCR)

Using the miRNeasy Serum/Plasma Kit (Qiagen, Gathersburg, MD, USA, 217184), total RNA was extracted as directed by the manufacturer. The Mir‐X miRNA qRT‐PCR TB Green Kit from Takara Bio Inc. in Japan was used for the RT‐qPCR procedure. SIRT1 and miR‐135a‐5p expression levels relative to U6 and ACTIN, respectively, were normalized. The primer sequences used were as follows: miR‐135a‐5p forward 5′‐GCCGCTATGGCTTTTTATTCCTATGTGA‐3′, U6 forward 5′‐GCTTCGGCAGCACATATACTAAAAT‐3′, SIRT1 forward 5′‐TATACCCAGAACATAGACACGC‐3′, reverse 5′‐CTCTGGTTTCATGATAGCAAGC‐3′, ACTIN forward 5′ ‐ TGACGTGGACATCCGCAAAG‐3′ and reverse 5′‐CTGGAAGGTGGACAGCGAGG ‐3′ primer. Relative expression was expressed as 2^−ΔΔCT^.

### 2.12 Dual‐luciferase reporter assay

To confirm the connection between SIRT1 and miR‐135a‐5p, a dual‐luciferase reporter test was carried out. Targetscan (https://www.targetscan.org/vert_80/), miDIP (http://ophid.utoronto.ca/mirDIP/index.jsp#r) and miRDB (https://mirdb.org/) were used to predict miR‐135a‐5p targets. The SIRT1 3′ untranslated region (3′UTR) gene fragment was created artificially, and wild‐type (WT) SIRT1 was used to create the complimentary sequence mutation site of the seed sequence. The pMIR reporter (Promega) was modified to include the endonuclease site. We created mutants with mutation sites (MUTs) binding to miR‐135a‐5p and a vector for the SIRT1 dual‐luciferase reporter gene. 293T cells in 96‐well plates were transfected with WT and MUT luciferase reporter plasmids using miR‐135a‐5p mimic plasmid/mimetic‐NC plasmid and Renilla vector (pRL‐TK; Promega). Cells were lysed using the Dual‐Luciferase Reporter Assay Kit (Promega) after 24 h of transfection. Each batch of cells was examined for the presence of Renilla luciferase (M2) and Firefly luciferase (M1) using the Dual‐Luciferase Reporter Assay Kit. The target gene's luciferase activity was expressed as M2/M1.

### 2.13 Wound‐healing assay

A total of 1 × 10^6^ transfected HTR8/SVneo cells were planted into each well of a 6‐well plate after 48 h of transfection, and the cells were grown to 95% confluence. The cell layers were punctured vertically with a 10 μL pipette tip. Using a light microscope (Nikon Corporation), the size of the wound area was measured at 0 and 24 h after the cells had been washed with PBS and grown in serum‐free media. Using ImageJ software, the relative wound area was assessed, and the mobility rate was computed. In triplicate, each experiment was carried out independently.

$$\text{Mobility rate}=\frac{\text{wound area}\;\mathrm{at}\;0\;h-\text{wound area}\;\mathrm{at}\;24\;h}{\text{wound area}\;\mathrm{at}\;0\;h\;}\times 100\%$$

### 2.14 Cell counting Kit‐8 assay

HTR‐8/SVneo cells were cultivated in 96‐well plates after 24 h of transfection, and the cell density was assessed at 0, 24, 48, 72 and 96 h. Each well containing serum‐free media received the CCK‐8 reagent (Sigma‐Aldrich) and was then incubated for 2 h. At a wavelength of 450 nm, absorbance was measured using an automatic microplate reader (Molecular Devices). Each experiment was carried out separately in triplicate while measuring the relative wound area.

$$\begin{array}{c} \text{Relative OD value}=\\ \;\text{OD value of experimental group}-\text{OD value of blank control} \end{array}$$

### 2.15 Transwell migration assay

The transwell migration was examined using transwell inserts made by Corning, Inc. called Costar. 600 μL of media without FBS was used to seed 1 × 10^5^ HTR‐8/SVneo cells into the transwell insert's upper chamber. The transwell plate's lower chamber received 600 μL of medium containing 10% FBS. The invading cells were incubated for 48 h at 37°C before being fixed for 15 min with 4% paraformaldehyde at room temperature and stained for 10 min with 2% crystal violet staining solution at room temperature. Utilizing an inverted microscope with a 200× magnification, the findings were obtained. After removing the non‐invading cells, the number of cells that made it through the Matrigel was counted. Using ImageJ (National Institutes of Health, version 1.8.0), cells in five random fields were counted.

### 2.16 Transwell invasion assay

Diluted Matrigel (BD Biosciences) was applied to Transwell inserts (Costar; Corning, Inc.) and incubated at 37°C for 1 h. 600 μL of media without FBS was used to seed 1 × 10^5^ HTR‐8/SVneo cells into the transwell insert's upper chamber. The transwell plate's lower chamber received 600 μL of medium containing 10% FBS. The invading cells were incubated for 48 h at 37°C before being fixed for 15 min with 4% paraformaldehyde at room temperature and stained for 10 min with 2% crystal violet staining solution at room temperature. Utilizing an inverted microscope with a 200× magnification, the findings were obtained. After removing the non‐invading cells, the number of cells that made it through the Matrigel was counted. Using ImageJ (National Institutes of Health, version 1.8.0), cells in five random fields were counted.

### 2.17 Statistical analysis

Statistical Package for Social Sciences (SPSS) for Windows, Version 20.0 (IBM Corp.) was used to analyse all quantitative data. The data collected from at least three independent experiments were averaged to determine the results, which were then reported as a mean and standard deviation (SD). Dunnett's test was used after Student's *t*‐test or one‐way analysis of variance (anova) to determine the significance of differences. The cutoff for statistical significance was *p* < 0.05.

## 3 RESULTS

### 3.1 Identification of differentially expressed miRNAs (DEMis)

**FIGURE 1:**
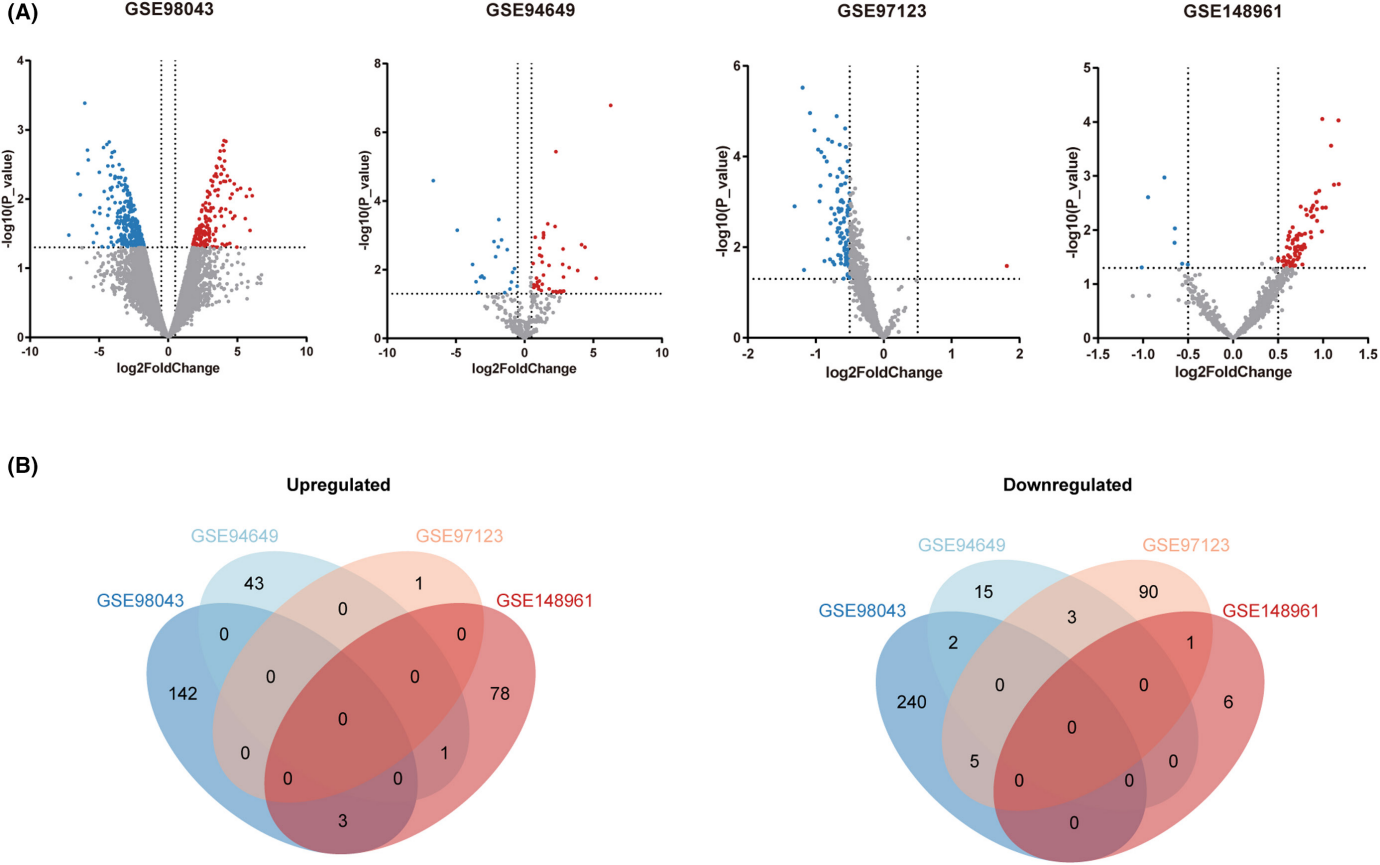
Volcano plots of differentially expression miRNAs (DEMis) and Venn diagrams of microarray data. (A) Volcano plots of GSE98043, GSE94649, GSE97123 and GSE148961 microarray data. Red and blue spots represent differentially expressed miRNAs (DEMis). Red represents upregulated miRNAs, and blue represents downregulated miRNAs. (B) Venn diagrams of upregulated and downregulated miRNAs in each microarray dataset.

The GEO database's GSE98043, GSE94649, GSE97123 and GSE148961 were chosen in accordance with the preceding inclusion criteria. Table 1 provides a list of the datasets' characteristics. Utilizing volcano plots and Venn diagrams, the DEMis of each dataset are displayed (Figure 1A,B). Candidate DEMis were found to be four upregulated and eleven downregulated common DEMis (Table 2).

**TABLE 1:** Features of enrolled GEO datasets.

GSE number	Platform	No. of controls	No. of patients	Upregulated DEMis	Downregulated DEMis
GSE98043	GPL21575	2	2	167	315
GSE94649	GPL18402	6	6	44	20
GSE97123	GPL17537	12	12	1	99
GSE148961	GPL25243	12	18	83	7

**TABLE 2:** Commonly regulated differentially expressed miRNAs (DEMis).

Upregulated	Downregulated
hsa‐miR‐135a‐5p hsa‐miR‐520e hsa‐miR‐1245b‐3p hsa‐miR‐188‐5p	hsa‐miR‐1972 hsa‐miR‐570‐3p hsa‐miR‐2278 hsa‐miR‐548‐5p hsa‐miR‐3131 hsa‐miR‐4707‐5p hsa‐miR‐3135B hsa‐miR‐4454 hsa‐miR‐106b‐5p hsa‐miR‐26b‐5p hsa‐miR‐548d‐3p

### 3.2 Functional annotation and pathway enrichment analysis

**FIGURE 2:**
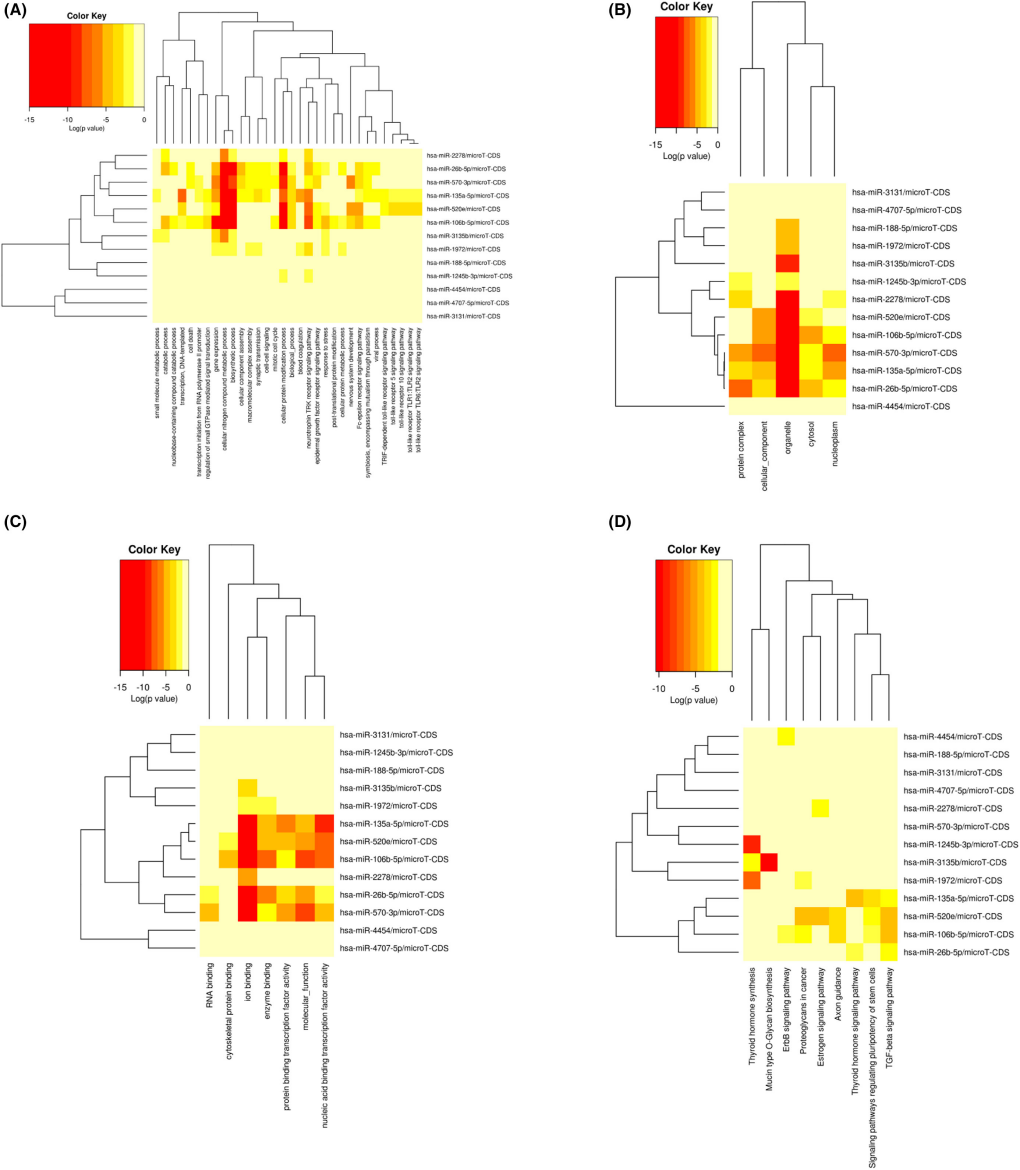
Significantly enriched Gene Ontology (GO) and KEGG pathway terms of differentially expression miRNAs (DEMis). (A) The cluster diagram shows 32 biological processes of effective enrichment. (B) The cluster diagram shows five cellular components of effective enrichment. (C) The cluster diagram shows seven molecular functions of effective enrichment. (D) The cluster diagram shows the KEGG pathway of effective enrichment. The ordinate represents the DEMi in the interaction network, and the abscissa represents the molecular functions of miRNA target gene enrichment. The gradual colour represents the log value (*p*‐value).

**FIGURE 3:**
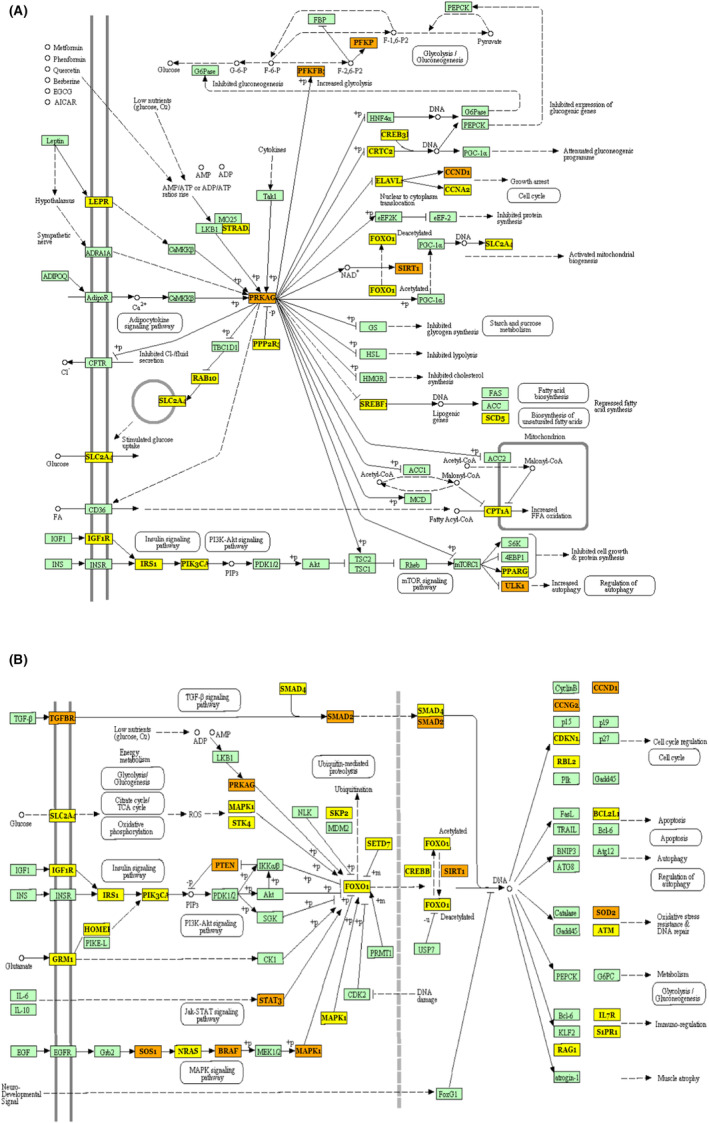
Signal pathway of AMPK and FOXO from DIANA mirPath v.3. (A) Signal pathway of AMPK from DIANA mirPath v.3. (B) Signal pathway of FOXO from DIANA mirPath v.3. Yellow represents the target genes, and orange represents that the target gene is enriched in more than one pathway.

In order to reflect the dynamic alteration processes during endothelial development, a Gene Ontology (GO) analysis was done on three distinct aspects: biological process (BP), cellular component (CC) and molecular function (MF) (Figure 2A–C). The following were the top three annotations, ranked by the *p*‐value for each aspect: CC: organelles (GO:0043226), cellular components (GO:0005575) and protein complexes (GO:0043234); MF: nucleic acid binding transcription factor activity (GO:0001071), molecular function (GO:0003674) and ion binding (GO:0043167). BP: cellular nitrogen compound metabolic process, biosynthetic process and cellular protein modification process. The most significantly enriched pathways for DEMis were next examined using a KEGG pathway enrichment analysis using DIANA mirPath v.3 (https://dianalab.e‐ce.uth.gr/html/mirpathv3/index.php?r=mirpath) (Figure 2D). Thyroid hormone synthesis (hsa04918), TGF‐signalling pathway (hsa04350) and mucin‐type O‐glycan biosynthesis (hsa00512) were the top three annotations rated by the *p*‐value in each aspect. The AMPK (hsa04152) and FOXO signalling pathways (hsa04068), as well as other significant GDM‐related pathways, were also elevated in these DEMi (Figure 3A,B). Orange was frequently used to mark SIRT1, PRKAG and CCND1, indicating that more than one route was enriched. SIRT1 was chosen for further analysis after being screened for relevance and significance in GDM. SIRT1 and the PI3K/AKT signalling pathway were shown to be regulated by one another in AMPK‐ and FOXO‐mediated transcription, suggesting that miR‐135a‐5p not only controls both the AMPK and FOXO signalling pathways but also controls the PI3K/AKT signalling system via SIRT1.

### 3.3 Clinical characteristics

Table 3 displays the clinical traits of the study subjects. Age, weight and history of the mother, the baby's gender and birth weight, the weight of the placenta, and the levels of total cholesterol (TC), high‐density lipoprotein (HDL) and low‐density lipoprotein (LDL) were not statistically different between the GDM and control groups. Women with GDM had substantially higher levels of fasting blood glucose (FPG), triglycerides (TG), apolipoprotein A1 (APOA1) and APOB/APOA1 than women in the control group (*p*‐value <0.05, *n* = 30/group).

**TABLE 3:** Baseline characteristics of women included in this analysis.

	Control *n* = 30	GDM *n* = 30	*p*‐Value
Age (years)	28.63 ± 0.5560	29.23 ± 0.5944	0.4640
Height (cm)	160.80 ± 0.9212	161.50 ± 1.138	0.6425
Pre‐pregnancy weight (kg)	52.96 ± 1.2800	58.44 ± 1.4340	0.0061
Pre‐pregnancy BMI (kg/m^2^)	20.48 ± 0.4402	22.39 ± 0.4766	0.0064
Weight at delivery (kg)	67.82 ± 1.6480	71.19 ± 1.4040	0.1246
BMI at delivery (kg/m^2^)	26.22 ± 0.6020	27.28 ± 0.4066	0.1488
Systolic pressure (mmHg)	117.20 ± 1.6030	118.90 ± 1.0690	0.3905
Diastolic pressure (mmHg)	73.50 ± 0.9220	74.90 ± 1.1930	0.3570
Gravidity history	2.13 ± 0.2075	1.77 ± 0.1639	0.1709
Parity history	1.57 ± 0.1143	1.43 ± 0.1038	0.3913
Gestational age (days)	276.50 ± 1.281	275.50 ± 1.217	0.5865
Neonatal sex (female) (%)	50.00	46.67	1.0000
Foetal weight (g)	3267.00 ± 64.06	3300.00 ± 74.39	0.7400
Placental weight (g)	585.20 ± 15.12	567.50 ± 15.74	0.4215
FPG (mmoL/L)	4.39 ± 0.1269	5.03 ± 0.1811	0.0051
TG (mmoL/L)	2.86 ± 0.1472	3.54 ± 0.2908	0.0414
TC (mmol/L)	6.40 ± 0.2256	6.16 ± 0.2221	0.4622
APOA1 (g/L)	1.65 ± 0.0592	1.83 ± 0.0504	0.0282
APOB (g/L)	1.23 ± 0.0662	1.13 ± 0.0423	0.2139
APOB/APOA1	0.76 ± 0.0463	0.63 ± 0.0315	0.0238
HDL (mmoL/L)	1.83 ± 0.0583	1.74 ± 0.0593	0.3168
LDL (mmoL/L)	3.59 ± 0.1813	3.27 ± 0.1414	0.1689

### 3.4 Plasma exosomes of GDM and normal pregnancy

**FIGURE 4:**
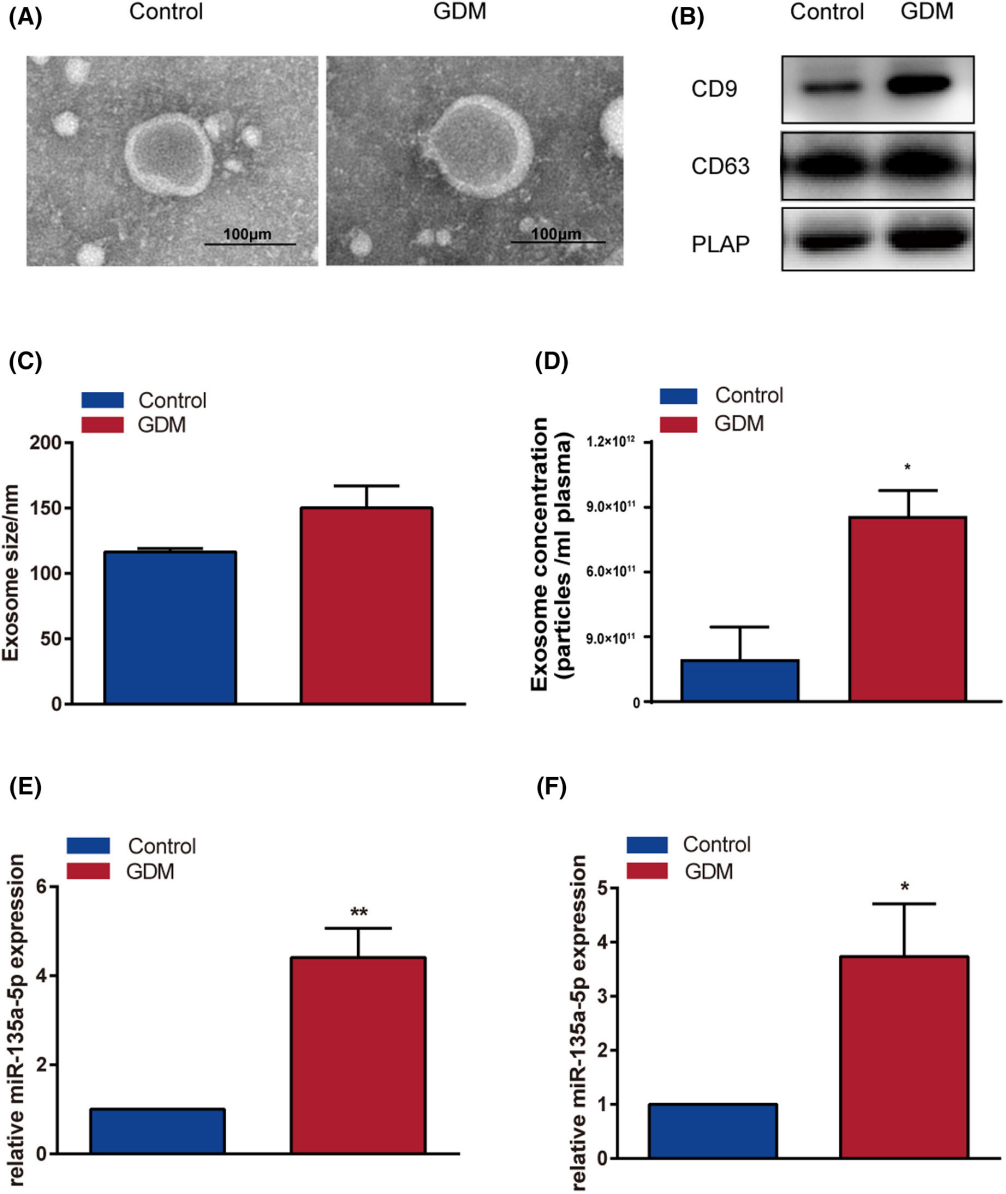
miR‐135a‐5p was abundantly expressed in placenta‐derived exosomes of GDM. (A) Transmission electron microscopy of plasma exosomes of each group. (B) Western blotting of plasma exosomes of each group: CD9, CD63 and PLAP. (C–D) The size and concentration of exosomes were detected using ZetaView PMX 110. (E) Expression of miR‐135a‐5p in placenta‐derived exosomes isolated from peripheral blood in the GDM and control groups. (F) Expression of miR‐135a‐5p in the placenta of GDM and control groups. *p < 0.05, **p < 0.01.

Western blot analysis for CD9, CD63 and PLAP was positive, and transmission electron microscopy demonstrated a cup‐shaped morphology (Figure 4A,B). Particles between 90 and 150 nm were discovered using nanoparticle tracking analysis. There was no statistically significant difference in the mean exosome diameter between the GDM and control groups (116.4 ± 2.706 nm vs. 150.3 ± 16.63 nm; *p*‐value = 0.1142, *n* = 3/group). However, the exosome concentrations of GDM groups were discovered to be significantly higher than that in the control groups (8.533e+011 ± 1.245e+011 vs. 1.897e+011 ± 1.554e+011 particles/mL plasma; *p*‐value <0.05, *n* = 3/group) (Figure 4C,D).

### 3.5 miR‐135a‐5p was abundantly expressed in placenta‐derived exosomes of GDM patients

The mRNA expression levels in the GDM and control groups were determined using RT‐qPCR. The expression levels of hsa‐miR‐135a‐5p were found to be considerably higher in the GDM group compared to the control group based on the chosen GEO datasets. The current analysis confirmed that miR‐135a‐5p in placenta‐derived exosomes isolated from peripheral blood was considerably elevated in the GDM group compared to the control group (Figure 4E). In comparison with the control group, miR‐135a‐5p expression in GDM placental tissue also showed higher expression (*p*‐value <0.05) (Figure 4F). These findings reveal that miR‐135a‐5p is substantially expressed in exosomes generated from the placenta of GDM patients.

### 3.6 miR‐135a‐5p promotes the proliferation, invasion and migration of the HTR‐8/SVneo cells

**FIGURE 5:**
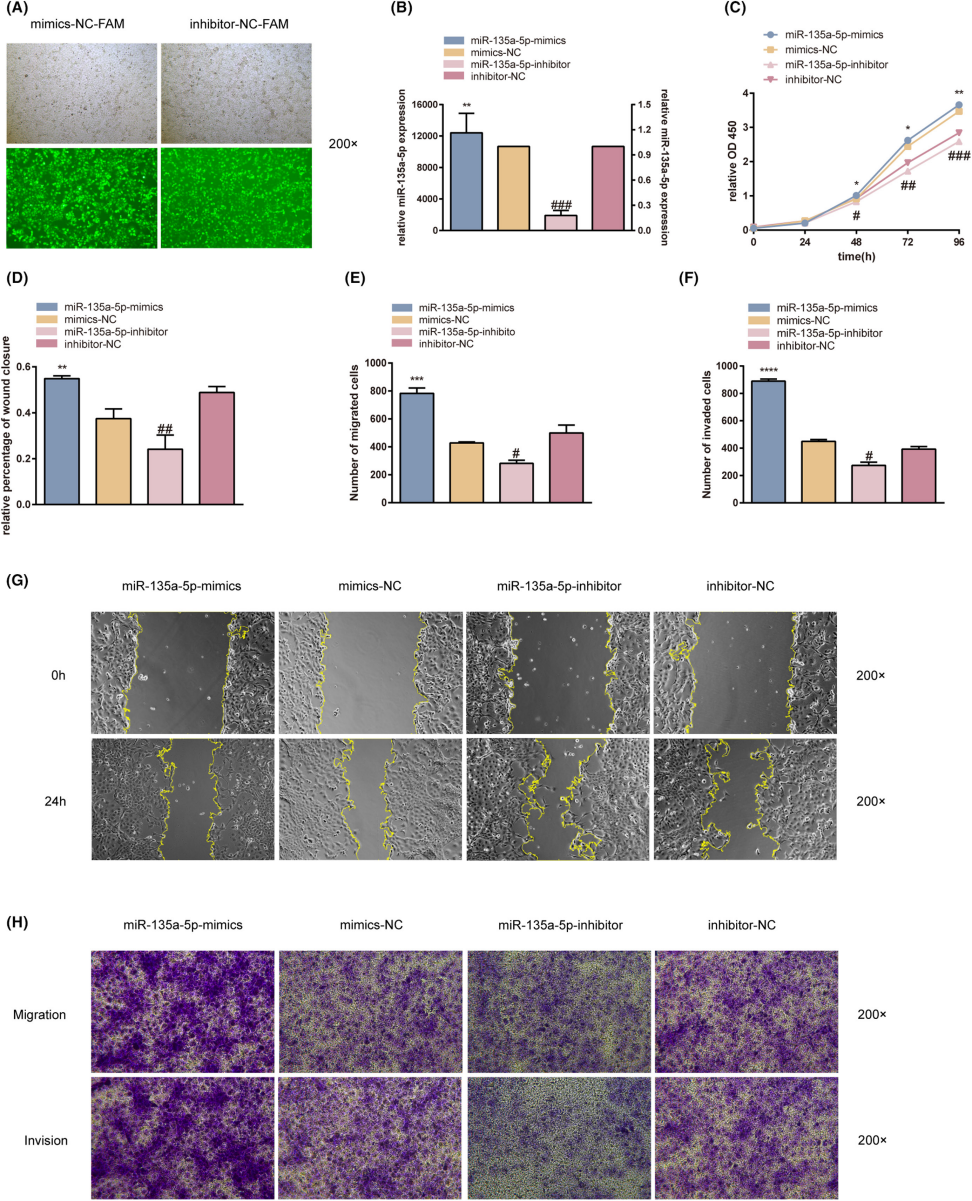
Transfection efficiency of HTR8/SVneo cells. (A) Transfection efficiency in mimics‐NC‐FAM and inhibitor‐NC‐FAM groups was observed using a fluorescent phase contrast inverted microscope group. (B) Transfection efficiency of miR‐135a‐5p in HTR8/SVneo cells was presented using RT‐qPCR. (C) The CCK‐8 assay was used to determine the effects of miR‐135a‐5p on the proliferation of HTR‐8/SVneo cells. (D–G) The wound‐healing assay was used to determine the effect of miR‐135a‐5p on the migration of HTR‐8/SVneo cells. (E, F and H) Transwell migration and invasion assay to determine the effect of miR‐135a‐5p on the migration and invasion of HTR‐8/SVneo cells. *p < 0.05, **p < 0.01, ***p < 0.001, ****p < 0.0001.

HTR‐8/SVneo cells transfected with miR‐135a‐5p‐mimics, miR‐135a‐5p‐inhibitors, mimics‐NC‐FAM and inhibitors‐NC‐FAM were grown to determine whether miR‐135a‐5p isolated from placental exosomes impacted the development of GDM. RT‐qPCR was used to confirm the effectiveness of the transfection after it had been examined using a fluorescent phase contrast inverted microscope (NIKON Eclipse Ts2R‐FL, Japan) (Figure 5A). When compared to the matching control group, the expression of miR‐135a‐5p was significantly higher in the mimic group and significantly lower in the inhibitor group (*p*‐value <0.01 and *p*‐value <0.001, respectively) (Figure 5B). The current investigation demonstrated that compared to the matching control group, cell proliferation, invasion and migration were greatly boosted in the mimic group and dramatically decreased in the inhibitor group (*p*‐value <0.05) (Figure 5C–H, Table 4). These findings suggest that miR‐135a‐5p encourages HTR‐8/SVneo cell proliferation, invasion and migration.

**TABLE 4:** Intercellular area and healing rate in each group.

	0 h	24 h	Healing rate
Mimics	7,759,800 ± 690,292	5,822,069 ± 661,323	0.5484 ± 0.01222
Mimics‐NC	8185295.4 ± 1,303,675	4,206,354 ± 898,727	0.3742 ± 0.04201
Inhibitors	8145075.4 ± 356,266	3,679,169 ± 296,337	0.2407 ± 0.06226
Inhibitors‐NC	8206768.6 ± 626,481	5156106.6 ± 1,022,687	0.4878 ± 0.02571

### 3.7 miR‐135a‐5p regulates SIRT1 expression via target binding

**FIGURE 6:**
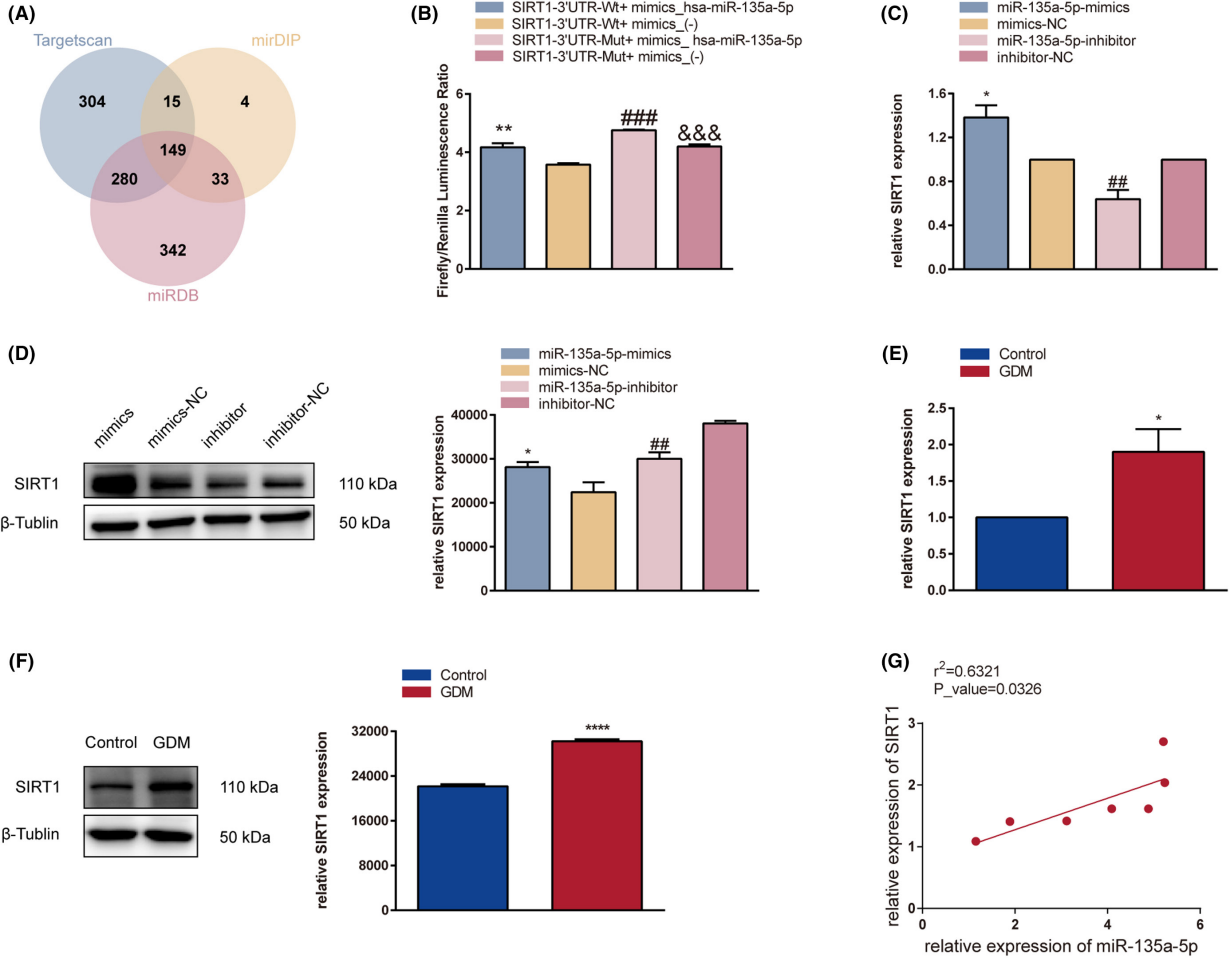
miR‐135a‐5p regulates SIRT1 expression via target binding. (A) Venn diagram depicting the downstream miR‐135a‐5p genes predicted using miDIP, Targetscan and miRDB databases. (B) The targeting relationship between hsa‐miR‐135a‐5p and SIRT1 was identified using the Dual‐Luciferase Reporter. HTR‐8/SVneo cells were co‐transfected with the wild‐type or mutant SIRT1 reporter plasmid and the miR‐135a‐5p mimics or mimics‐NC. (C) RT‐qPCR analysis of SIRT1 mRNA expression in HTR‐8/SVneo cells after transfection. (D) Western blotting analysis of SIRT1 protein expression in HTR‐8/SVneo cells after transfection. (E) RT‐qPCR analysis of SIRT1 mRNA expression in placental tissue. (F) Western blotting analysis of SIRT1 protein expression in placenta tissue. (G) Pearson's correlation analysis of miR‐135a‐5p and SIRT1 expression in placental tissues. *p < 0.05, **p < 0.01, ***p < 0.001, ****p < 0.0001.

The miRNA target gene prediction websites mirDIP, Targetscan and miRDB were used to identify has‐miR‐135a‐5p targets, and the 3′UTR of human SIRT1 demonstrated a highly conserved binding site for miR‐135a‐5p (Table 5). This information was used to further investigate the potential mechanisms of miR‐135a‐5p and its potential downstream genes. 513 genes were discovered and are shown in a Venn diagram of the intersecting has‐miR‐135a‐5p genes (Figure 6A). The AMPK signalling pathway was one of the primary signalling pathways regulated by miR‐135a‐5p, according to the previous KEGG enrichment analysis; SIRT1, PFKP, PFKFB2, PRKAG, CCND1 and ULK1 were tagged with orange, indicating that more than one pathway was enriched, of which SIRT1 was the most frequent and significant gene. This binding interaction was examined using a luciferase reporter experiment. In comparison with the mimic‐NC and 3′UTR (MUT) + mimics_hsa‐miR‐135a‐5p groups, the 3′‐UTR (WT) + mimics_hsa‐miR‐135a‐5p group's luciferase activity was significantly higher (*p*‐value <0.01 and *p*‐value <0.001, respectively) (Figure 6B). By using RT‐qPCR and WB in HTR‐8/SVneo cells and the placenta, the results demonstrated the targeting link between hsa‐miR‐135a‐5p and SIRT1 (*p*‐value <0.05 and *p*‐value <0.01, respectively) (Figure 6C,D). When compared to the mimics‐NC group, the miR‐135a‐5p‐mimics group dramatically enhanced SIRT1 expression, while the miR‐135a‐5p‐inhibitor group considerably decreased SIRT1 expression. Comparing the GDM placenta group to the control group, SIRT1 expression was considerably increased (*p*‐value <0.05) (Figure 6E,F). In HTR‐8/SVneo cells, the expression of miR‐135a‐5p was positively linked with the expression of SIRT1 mRNA according to Pearson's correlation analysis (*r* ^2^ = 0.6321, *p*‐value <0.05) (Figure 6G). The findings above show that miR‐135a‐5p targets SIRT1 and that miR‐135a‐5p encourages SIRT1 expression.

**TABLE 5:** miRNA target gene prediction of has‐miR‐135a‐5p and SIRT1.

	Position 358–364 of SIRT1 3′UTR	Has‐miR‐135a‐5p
Predicted paring sequence	3′…U ***AUGGCUU*** UUUAUUCCUAUGUGA…5′	5′AA ***AAGCCAT*** CGGAAT3′
Site type	Conserved 7mer‐m8 sites	
Cumulative weighted context++ score	−0.25	
Total context++ score	−0.25	
Aggregate PCT	0.63	

### 3.8 miR‐135a‐5p targeting SIRT1 promotes PI3K/AKT pathway activity to promote GDM development

**FIGURE 7:**
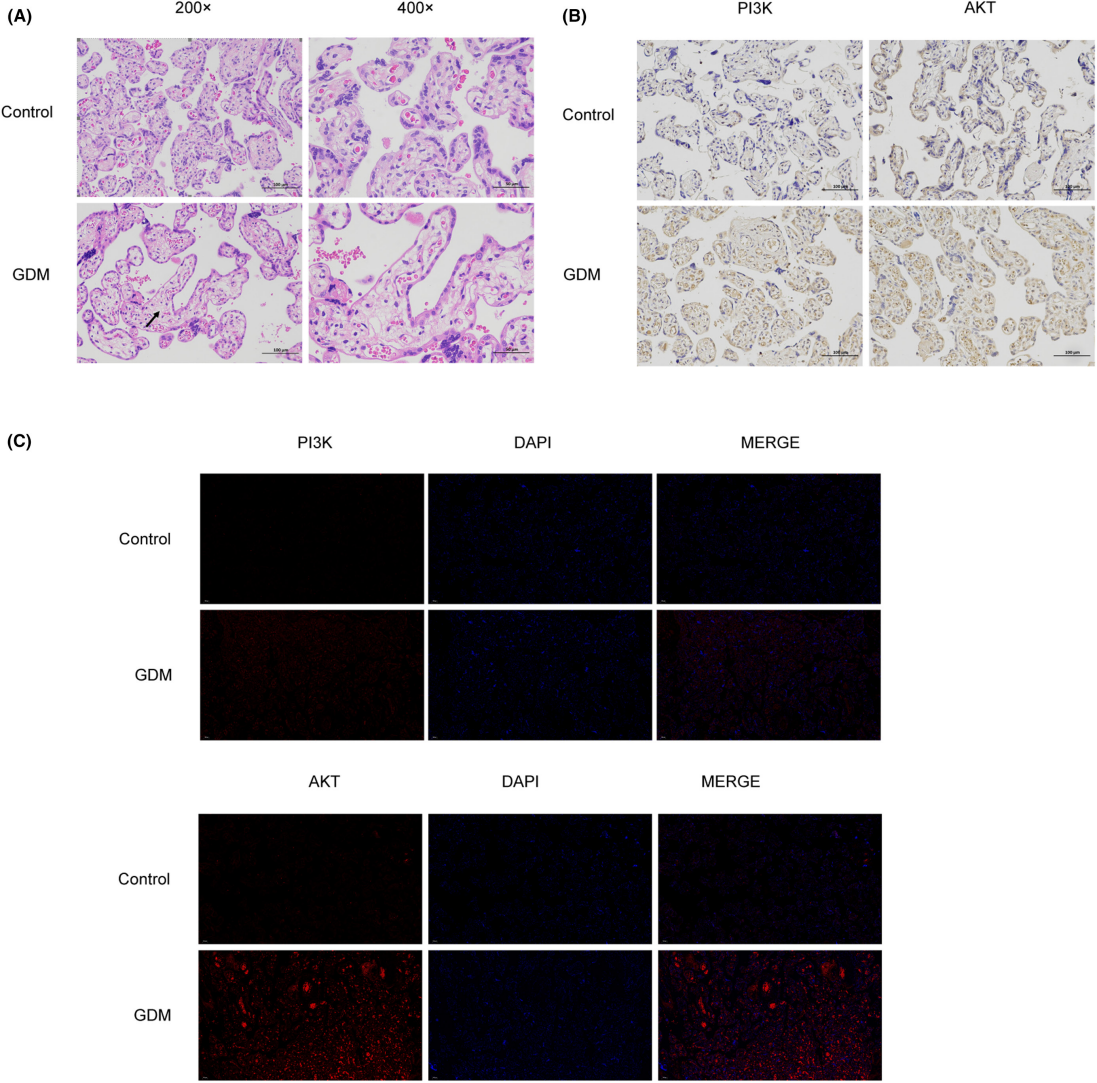
miR‐135a‐5p targeting SIRT1 promotes PI3K/AKT pathway activity to initiate GDM development. (A) HE staining to determine pathological changes of placental tissue. (B) Representative images of PI3K and AKT protein expression in placenta tissues detected via immunohistochemical staining. (C) Representative images of PI3K and AKT protein expression in placenta tissues detected via immunofluorescence.

**FIGURE 8:**
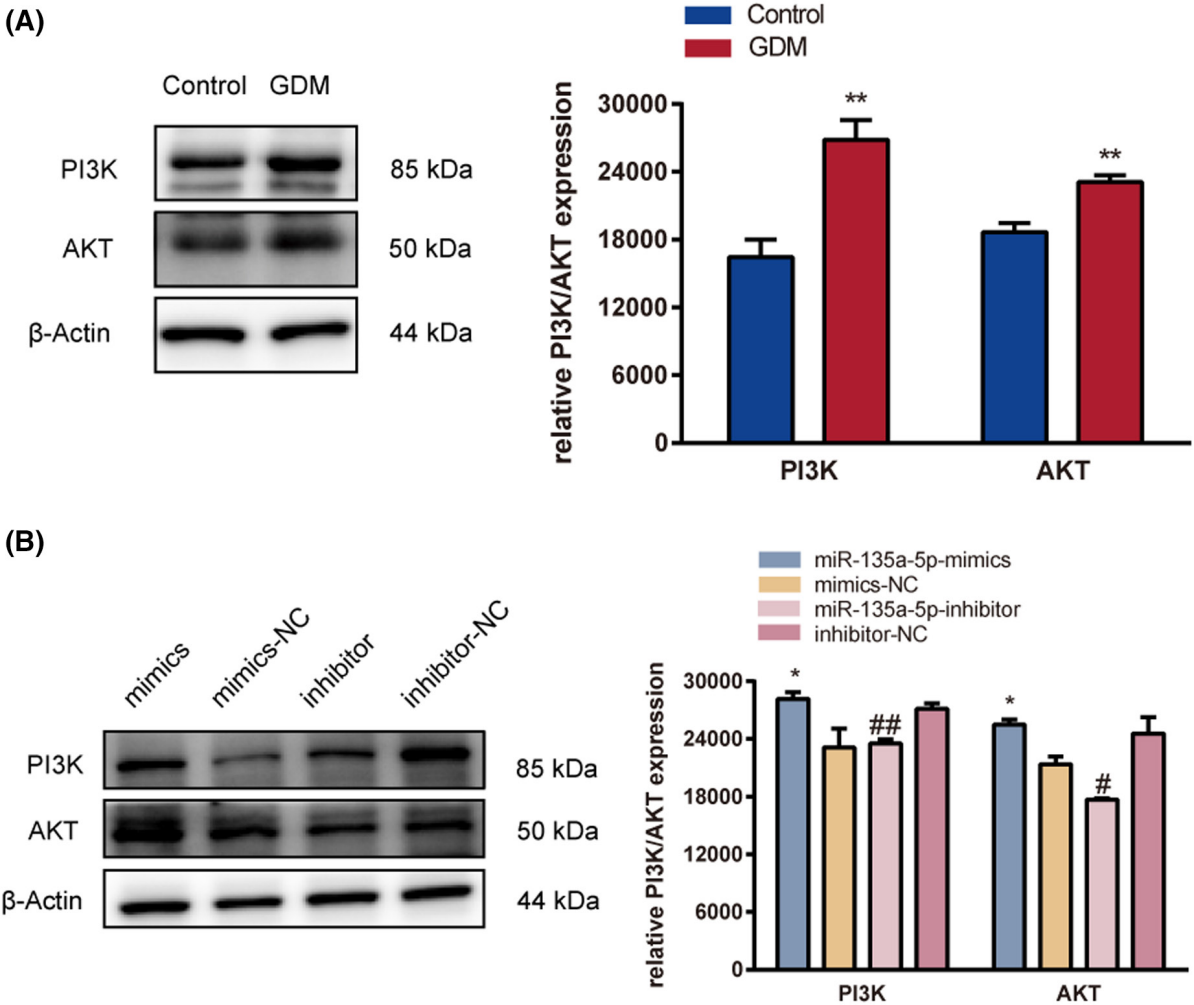
miR‐135a‐5p targeting SIRT1 promotes PI3K/AKT pathway activity to initiate GDM development. (A) Western blotting analysis of PI3K and AKT protein expression in placental tissues. (B) Western blotting analysis of PI3K and AKT protein expression in cells. *p < 0.05, **p < 0.01.

Previous research has demonstrated that SIRT1 has a role in a number of biological functions in human chorionic trophoblast cells, including trophoblast migration, proliferation and invasion.^10^ Pathological alterations in the placental tissue were seen to confirm this finding (Figure 7A). The number of capillaries in the villi was smaller in the GDM group than in the control group, and a significant portion of the villi were swollen and had an increased volume. The expression of PI3K and AKT was clearly higher in the GDM group than in the control group, as shown by immunohistochemical staining and immunofluorescence methods (Figure 7B,C). The findings indicate that miR‐135a‐5p enhances PI3K/AKT pathway activity, which was confirmed by western blotting in HTR‐8/SVneo cells and the placenta (*p*‐value <0.05) (Figure 8A,B). While PI3K and AKT expression was significantly higher in the miR‐135a‐5p‐mimics group than in the mimics‐NC group, it was significantly lower in the miR‐135a‐5p‐inhibitor group as compared to the inhibitor‐NC group. These results demonstrate the potential role of miR‐135a‐5p in GDM via enhancing PI3K/AKT signalling pathway activity in HTR‐8/SVneo cells.

## 4 DISCUSSION

GDM is the most common pregnancy metabolic complication, with a worldwide incidence of approximately 14% according to the International Diabetes Federation (IDF).^11^ Both mothers and foetuses with GDM are more likely to suffer from various short‐ and long‐term adverse consequences than those of normal pregnancies. Nevertheless, the genetic and environmental variety identified in mechanistic and epidemiological investigations plays a role in the complicated aetiology of GDM. Many of the mechanisms underlying GDM are not unique to GDM and encompass the common pathology of other diseases such as T2D, PCOS and cardiovascular disease.^12^ Therefore, correlated pathways need to be discovered, which could potentially shed light on GDM development, prevention and treatment. As mentioned above, with the advancing knowledge of exosomes, it has been identified that exosomes are promising agents for predicting, diagnosing and treating GDM.^13^ Exosomal components are actively secreted towards diverse cells and participate in intercellular communication; thus, exosomal miRNAs are considered prominent mediators for enhancing the understanding of the intricate molecular mechanism of GDM in the future.^14^ The placenta is a specific interface between maternal and foetal circulation, where a variety of molecular exchanges occur through extracellular vesicles, especially via exosomes.^15^ Notably, placenta‐derived exosomes are differentiated from other exosomes by the presence of placenta‐specific miRNAs or proteins, such as placental alkaline phosphatase (PLAP) and human leukocyte antigen G (HLA‐G).^16^ In general, quantification of placenta‐derived exosomal miRNAs in maternal peripheral blood reflects foetal growth and shows a novel perspective in deciphering the mechanisms underlying pregnancy‐relevant diseases. In this study, GDM‐related differentially expressed miRNAs (DEMis) were screened and linked to possible signalling pathways.^14^ We investigated the potential impact of exosomal miR‐135a‐5p from the placenta on GDM. Our findings showed that exosomal miR‐135a‐5p can target SIRT1 to enhance the PI3K/AKT signalling pathway and control the development of GDM. These findings, which are pertinent to GDM, show that exosomes mediate a wide range of diagnostic and treatment efficacies for diverse illnesses.

Previously, miR‐135a‐5p was mainly discussed in the context of functional disability in cancer, neurogenic diseases and cardiovascular diseases.^17^, ^18^, ^19^ However, miR‐135a‐5p may also contribute to inflammation, adipogenesis and glucose metabolism, implying its potential for further mechanical exploration of GDM.^20^, ^21^, ^22^ It is still unknown how miR‐135a‐5p affects the development of GDM. This work demonstrated that miR‐135a‐5p is a target gene for placenta‐derived exosomes that influence the onset and progression of GDM. We discovered that GDM placentas' exosomes can spread miR‐135a‐5p to HTR‐8/SVneo cells, encouraging their growth, invasion and migration.

Sirtuins are members of the Sir2 (silent information regulator 2) family, which consists of nicotinamide adenine dinucleotide (NAD+)‐dependent protein deacetylases and ADP‐ribosyltransferases.^23^ Among the seven different mammalian sirtuins, SIRT1 is the most well characterized Sir2 family member and is expressed mainly in the nucleus.^24^ SIRT1 is a prototype mammalian NAD(+)‐dependent protein deacetylase, emerging as a key metabolic sensor in various metabolic tissues.^25^ Due to its numerous roles, the PI3K/AKT pathway is still worthwhile of investigation. The PI3K/AKT pathway plays a central role in various physiological processes such as glucose homeostasis, lipid metabolism, protein synthesis, and cell proliferation and survival.^26^, ^27^, ^28^, ^29^ It is believed that damage to the PI3K/AKT pathway in various tissues results in metabolic diseases, such as obesity and type 2 diabetes, leading to insulin resistance that occurs in these diseases and exacerbates the PI3K/AKT pathway, causing a vicious circle.^27^ It is still unclear how Sirtuins and the PI3K/AKT signalling pathway are related to metabolic illnesses like GDM, despite advances in our understanding of their regulation and functions. In physiological terms, insulin binds to the insulin receptor to activate PI3K via phosphorylation, and PI3K, in turn, transforms PIP2 into PIP3, activating AKT through phosphorylation to maintain a balance in the metabolism of blood glucose and lipid. Accordingly, we demonstrated that miR‐135a‐5p delivered by placental exosomes targeted SIRT1 and disrupted GDM‐induced cellular dysfunction via activating the PI3K/AKT signalling pathway. Unfortunately, the current study lacks the necessary experiments to substantiate the phosphorylation in the PI3K/AKT signalling pathway.

The results of this study indicated that placenta‐derived miR‐135a‐5p increases the proliferation, invasion and migration of placental trophoblast cells by targeting SIRT1. This miRNA also promotes the function of the PI3K/AKT signalling pathway. In addition to demonstrating the critical function of miR‐135a‐5p in GDM, this study also demonstrated its relationship to SIRT1 and the PI3K/AKT signalling pathway. Nevertheless, if related animal tests were carried out, the results' veracity would be increased. Last but not least, the mechanical investigation of many diseases aims to expose their pathological processes for use in clinical diagnosis or treatment. Despite the intricacy of GDM, we offer a promising therapeutic approach and anticipate providing more chances to improve the health of both expectant mothers and foetuses.

## AUTHOR CONTRIBUTIONS

**Qiuyu Zhang:** Conceptualization (lead); data curation (lead); formal analysis (lead); funding acquisition (equal); investigation (lead); methodology (lead); project administration (lead); resources (lead); software (lead); visualization (equal); writing – original draft (lead); writing – review and editing (lead). **Xu Ye:** Conceptualization (supporting); data curation (supporting); formal analysis (supporting); investigation (supporting); methodology (supporting); project administration (supporting); resources (supporting); software (supporting); supervision (supporting); validation (supporting); visualization (supporting); writing – original draft (supporting); writing – review and editing (supporting). **Xia Xu:** Conceptualization (supporting); data curation (supporting); formal analysis (supporting); investigation (supporting); methodology (supporting); project administration (supporting); resources (supporting); software (supporting); writing – original draft (supporting); writing – review and editing (supporting). **Jianying Yan:** Conceptualization (supporting); data curation (supporting); formal analysis (supporting); funding acquisition (lead); investigation (supporting); methodology (supporting); project administration (supporting); resources (supporting); software (supporting); supervision (equal); validation (equal); visualization (equal); writing – original draft (supporting); writing – review and editing (supporting).

## FUNDING INFORMATION

This research was financially supported by the Guide Fund for the Development of Local Science and Technology from the Central Government (2020L3019), Joint Funds for the Innovation of Science and Technology, Fujian Province (2020Y9134), Fujian Provincial Health Technology Project (2021CXA034), National Health and Family Planning Commission Science Foundation (2019‐WJ‐04) and the Health Research Project of the Department of Finance (Fujian Finance refers to [2019] No. 827) (2020Y183).

## CONFLICT OF INTEREST STATEMENT

The authors declare that they have no known competing financial interests or personal relationships that could have appeared to influence the work reported in this paper.

## Data Availability

The original contributions presented in the study are included in the article. Further inquiries can be directed to the corresponding author.
